# Association for the Oral Instruction of the Deaf and Dumb

**Published:** 1903-08-08

**Authors:** 


### ASSOCIATION FOR THE ORAL INSTRUCTION OF THE DEAF AND DUMB.

The annual meeting of this Association was held at 11 Fitzroy Square on July 29th. There was a good attendance in spite of the very wet afternoon.

The Rev. C. H. Parez, who took the chair, moved the adoption of the report and said that the pupils trained at the Normal School, established by the Association, were enabled to go about the world as freely as those in full possession of all their senses and could see what people said even at a distance. A great effort had been made in the past year to bring the institution out of the rut in which it had stuck. It required a superhuman effort to make it move on, but they possessed an individual of gigantic enterprise in the person of their director, Mr. Van Praagh, who had put his shoulder to the wheel and had induced friends to give help to such an extent that the institution was now in a better position than it was this time last year. A certain sum of money had been collected which would enable them to carry on the work for some little time longer. They therefore hoped that during that time public opinion would be invigorated, the importance of giving the deaf proper instruction would be recognised, and they would receive help from public sources.

Mr. B. St. John Ackers, who seconded the report, said that this was the first institution to introduce into this country the system of oral instruction of the deaf and dumb, and therefore had a special claim upon all those who desired that the best method should become universal. There were very hopeful signs that the general public and teachers were more inclined to the pure oral system than ever before. He congratulated the Association on the success of their festival dinner fund, but wished the amount raised could have been doubled. It was no use trying to starve the teaching of the deaf; it was an expensive system, and if it was to equal the teaching of ordinary children much more must be given for its support. Not one sixpence was given by the Government for the training of teachers for the deaf, although 75 per cent. was given by Government towards the cost of training of the ordinary teachers. The want of education for the deaf was, however, far greater than for ordinary children.

Mr. Van Praagh said that looking back on the last 33 years he thought the association had every reason to be grateful for the work it had done. Unfortunately, the work had not met with the support it ought to have received. Their students were doing excellent work in the colonies and in India, as well as in the United Kingdom. The benefits they conferred on the deaf were not recognised by Government, but he hoped that new legislation would make up for the deficiencies of the past.

A most interesting exhibition was then given by pupils in various stages of instruction, beginning by a tiny boy who had only been learning for six weeks, but who could already articulate vowel sounds, and concluded by students who had completed their course, and who spoke in such natural voices and were so quick and correct at lip-reading that it was difficult to discern any difference between them and those endowed with all their senses. Prizes were then distributed by Miss Amy Paget.

